# Foreign Cry1Ab/c Delays Flowering in Insect-Resistant Transgenic Rice via Interaction With Hd3a Florigen

**DOI:** 10.3389/fpls.2021.608721

**Published:** 2021-02-11

**Authors:** Jianmei Fu, Guoqiang Liu, Biao Liu

**Affiliations:** ^1^Nanjing Institute of Environmental Sciences, Ministry of Ecology and Environment, Nanjing, China; ^2^Institute of Plant Protection, Jiangsu Academy of Agricultural Sciences, Nanjing, China; ^3^College of Life Sciences, Nanjing Agricultural University, Nanjing, China

**Keywords:** Cry1Ab/c protein, delayed flowering, E3 ubiquitin ligases, florigen protein Hd3a, *Hd3a* expression, protein interactions

## Abstract

Genetic modifications in rice, which resulted in insect resistance, have been highly efficacious. However, they have also induced undesirable secondary phenotypes, such as delayed flowering. The molecular mechanisms associated with these unwanted effects remain unclear. Here, we showed that the flowering time for insect-resistant transgenic *cry1Ab/c* rice Huahui-1 (HH1) was delayed, compared with that for the parental rice Minghui-63 (MH63), cultivated on farmland and saline–alkaline soils. In contrast, the insect-resistant transgenic *cry1C*^^*^ rice cultivars T1C-19 and MH63 had similar flowering times under the same conditions. We quantified the following: the expression of five major flowering genes in HH1, T1C-19, and MH63; florigen Hd3a protein expression levels in HH1 and MH63; interactions between Cry1Ab/c and the five main flowering proteins; and the effects of E3s ubiquitin ligase-mediated Cry1Ab/c expression on florigen Hd3a. Hd3a transcription was significantly lower in HH1 but not in T1C-19, compared with that in MH63. The results of yeast two-hybrid, complementary bimolecular fluorescence, and co-immunoprecipitation assays revealed that florigen Hd3a interacted with the exogenous Cry1Ab/c expressed in HH1 and not the exogenous Cry1C^^*^ expressed in T1C-19. When Cry1Ab/c, Hd3a, and E3s fusion proteins were transiently co-expressed in tobacco cells, the Hd3a expression level was significantly lower than the level of Cry1Ab/c and Hd3a co-expression. Thus, the downregulation of *Hd3a* expression and the interaction between Cry1Ab/c and Hd3a interfere with Hd3a protein expression and might cooperatively delay HH1 flowering time. To the best of our knowledge, this study is the first to explain the delay in flowering time in insect-resistant transgenic rice, mediated by interactions between exogenous and endogenous proteins. This information might help elucidate the molecular mechanisms associated with these unwanted phenotypes effects and improve the process of biosafety assessment of transgenic rice.

## Key Message

Here, we identified *cy1Ab/c*, the foreign gene expressed in transgenic rice. We confirmed that it binds and neutralizes florigen Hd3a and clarified the signaling pathway involved in the process. This information may help breeders develop innovative, high-yielding, stress-tolerant rice strains.

## Introduction

Currently, *Bt* from *Bacillus thuringiensis* is the most commonly used insecticidal gene worldwide (ISAAA, 2017). *Bt* encodes safe and efficacious crystalline (cry) insecticidal proteins (Cry toxins). They have specific insecticidal activity against Strongylida, Lepidoptera, Coleoptera, and Diptera insects. Therefore, Cry toxin genes have been widely used to construct genetically modified crops to enhance the resistance of crops to target insects. Genetic engineering has enabled us to achieve insect control in rice *via* the introduction of *Bt* into the rice genome. Research on transgenic rice has progressed rapidly and several transgenic insect-resistant rice varieties have been successfully designed over the last 20 years. These include KMD, Bt-shanyou63 (Bt-SY63), Huahui-1 (HH1), T1C-19, and T2A-1 (Tu et al., 1998, 2000; Ye et al., 2001; Chen et al., 2005; Tang et al., 2006). On October 22, 2009, the Ministry of Agriculture of China issued biosafety certificates permitting the cultivation of the transgenic insect-resistant rice lines HH1 and *Bt* Shanyou 63 (Bt-SY63) in Hubei Province. The Ministry renewed these permissions in December 2014 (Xia et al., 2011). Hence, HH1 and Bt-SY63 might undergo large-scale environmental release and be produced commercially in the future.

Although target insect resistance traits were established for certain rice cultivars, unintended effects may nonetheless occur, following the introduction of the exogenous *Bt* gene into the rice genome. This may adversely affect the intrinsic physiological metabolism (Jiang et al., 2018; Ling et al., 2018) or alter the agronomic traits (Shu et al., 2002; Chen et al., 2006; Kim et al., 2008; Xia et al., 2010) of the parental rice lines. However, the molecular mechanisms underlying these undesirable effects are unknown. Potential causes may include (1) host gene disruption or DNA sequence rearrangement at foreign gene insertion sites (Forsbach et al., 2003), (2) somatic variations during transgenic strain construction (Labra et al., 2001; Noro et al., 2007), (3) excess host cell biomass and energy consumption during foreign gene expression (Zeller et al., 2010), and (4) interactions between exogenous and endogenous proteins (Fu and Liu, 2020a). Currently, however, there is no direct empirical evidence to support these hypotheses.

Flowering time is an important agronomic trait in rice, as it determines grain yield and regional adaptation ability. Previous studies identified the following main rice flowering genes: *Hd3a* (Heading date 3a), *Hd1* (Heading date 1), *Hd6* (Heading date 6), *Ehd1* (Early heading date 1), and *Ghd7* (Heading Date7) (Yano et al., 2000; Takahashi et al., 2001; Kojima et al., 2002; Xue et al., 2008; Cho et al., 2016). The independent pathways Hd1–Hd3a and Ghd7-Ehd1-Hd3a control rice flowering time. Du et al. (2017) reported that Hd1 regulates Hd3a expression and promotes flowering under short-day conditions but inhibits it under long-day conditions. Tsuji et al. (2011) found that Ghd7 inhibits Ehd1 and Hd3a transcription, but did not inhibit Hd1 transcription. Thus, rice phenotypes exhibiting delayed flowering are created. The genes regulating rice flowering might eventually act on the Hd3a florigen protein, which is an integrated signal that moves from the leaf to the shoot apical meristem and induces flowering in rice. Therefore, it resembles the FT protein observed in *Arabidopsis* (Tamaki et al., 2007).

In the present study, we used two different insect-resistant transgenic rice strains, HH1 (harboring foreign *cry1Ab/c*) and T1C-19 (harboring foreign *cry1C*^^*^ with low sequence homology with foreign *cry1Ab/c*, and the similarity of their amino acid sequences was 45.1%), with the same genetic background as the materials used for research, and found that the flowering time of transgenic HH1 but not T1C-19 rice was delayed, compared with that of parental MH63 rice. *Hd3a* expression was significantly lower in HH1 than in MH63, but was not lower in T1C-19. To determine how Cry1Ab/c participates in HH1 flowering, we examined its interactions with the regulators of the flowering process, such as Hd3a, Hd1, Hd6, Ghd7, and Ehd1, using yeast two-hybrid, bimolecular fluorescence complementation (BiFC), and co-immunoprecipitation assays (co-IP), and determined the effects of these interactions on Hd3a florigen protein *via* western blotting. Foreign Cry1Ab/c protein expressed by HH1 interacted with Hd3a, whereas the foreign Cry1C^^*^ expressed by T1C-19 did not interact with it. In the former case, the Hd3a protein was downregulated *via* the 26S proteasome pathway, in a process mediated by E3 ubiquitin ligases. The findings herein may provide direct evidence showing that the downregulation of *Hd3a* expression and the interaction between Cry1Ab/c and Hd3a target the E3 ubiquitin ligase pathway for Hd3a degradation and cooperatively regulate flower signaling in transgenic HH1 rice. This would provide an empirical foundation for the identification and quantification of the signaling mechanisms by which the genetic modification of rice for superior disease or pest resistance results in untoward phenotypic effects.

## Materials and Methods

### Plant Materials and Growth Conditions

Insect-resistant transgenic *cry1Ab/c* rice HH1, transgenic *cry1C*^^*^ rice T1C-19, and non-transgenic Minghui-63 (MH63) were used in this study. The 1,830-bp-long *cry1Ab/c* transgene was synthesized from the 1,344-bp-long *cry1Ab* gene (GenBank accession no. X54939) and the 486-bp-long *cry1Ac* gene (GenBank accession no. Y09787) and driven by the rice actin1 promoter (Tu et al., 1998). The *cry1C*^^*^ gene was synthesized and optimized using the wild-type *cry1Ca5* gene of *B. thuringiensis*. The 2,300-bp-long modified *cry1C*^^*^ sequence shares 84% nucleotide sequence homology with wild-type *cry1Ca5*. HH1 and T1C-19, which had the same genetic background, exhibited high δ-endotoxin expression and insecticidal activity against the borers *Chilo suppressalis* and *Tryporyza incertulas*. The rice lines were provided by the National Key Laboratory of Crop Genetic Improvement, at Wuhan, China (Tu et al., 1998; Tang et al., 2006).

The *Nicotiana benthamiana* used in this study was cultivated in a greenhouse under a 16-h:8-h light:dark photoperiod, at 22–25 and 18–22°C under light and dark conditions, respectively. After 4–6 weeks, the seedlings reached the five-leaf stage and were used in *Agrobacterium*-mediated transient transformation experiments.

### Flowering Time Analysis

To measure the total days to flowering, rice seedlings were grown on farmland and saline–alkaline soils, as described previously (Fu et al., 2018; Fu and Liu, 2020b). Briefly, the rice lines with consistent growth from a small breeding plate were transplanted into large pots (840 mm length × 560 mm width × 360 mm height) filled with saline–alkali soil or farmland soil at the five-leaf stage. Ten replicate pots were set up for each treatment, and 12 seedlings arranged in four rows and three columns at 19–21 cm intervals were uniformly distributed in each replicate pot; in total, 720 plants were randomly placed in the glasshouse. Fifty rice plants were randomly selected per treatment to record the total days to flowering. The appearance of the first spike in each plant was recorded as the flowering of the plant, and the day at which the earliest flowering plants appeared was determined to be the first day. Then, the flowering time of other plants was recorded every day until all the plants were flowering.

### RNA Preparation and RT-qPCR

At ∼30 days before flowering, we collected leaf and shoot apex samples from HH1, T1C-19, and MH63. Total RNA was extracted and purified with the RNeasy Plant Mini kit (Qiagen, Hilden, Germany). One microgram of total RNA was reverse-transcribed into cDNA with a PrimeScript RT-PCR kit (TaKaRa Bio Inc., Kusatsu, Shiga, Japan), according to the manufacturer’s instructions. We designed 100–300-bp-long quantitative primers and established the amplification efficiency of each primer pair by diluting cDNA samples 10-fold and preparing five dilution gradients. Primer amplification efficiency was in the range of 95–105% (Supplementary Table 1). The qRT-PCR assays were performed with a TB Green^TM^ Premix Ex Taq^TM^ kit (TaKaRa Bio Inc., Kusatsu, Shiga, Japan), according to the manufacturer’s protocol, and run in a LightCycler^®^ 480 II real-time system (Roche Diagnostics, Basel, Switzerland). Data were calibrated against the Ct values of stably expressed rice β-actin. Target gene expression was characterized by the 2^–Δ^ ^Δ^ ^Ct^ method (Schmittgen and Livak, 2008). Five samples were pooled per plot and five biological replicates were randomly selected per experiment.

### Detection of Exogenous Protein Expression by Western Blotting or Enzyme-Linked Immunosorbent Assay

To detect Cry1Ab/c expression in HH1, the total protein content was extracted from HH1 and MH63 leaves grown on farmland and saline–alkaline soil at the heading stage. NP-40 lysate (P0013F; Beyotime Biotechnology, Shanghai, China) was used to extract leaf proteins, according to the manufacturer’s instructions. The rice leaves were pulverized in liquid nitrogen and 1 ml NP-40 lysate was added in 1 mM phenylmethysulfonyl fluoride (PMSF) protease inhibitor to the powder. The samples were vortexed, mixed, and centrifuged at 13,000 × *g* and 4°C for 10 min. The supernatants were transferred to new Eppendorf tubes (EP; Eppendorf, Hamburg, Germany) for sodium dodecyl sulfate-polyacrylamide gel electrophoresis (SDS-PAGE) and western blot analysis. To detect the target Cry1Ab/c protein, the monoclonal Cry1Ab anti-mouse antibody (Abcam, Cambridge, United Kingdom) was used for hybridization at a 1:1,500 dilution. β-Actin (1:1,500; Sangon Biotech, Shanghai, China) was used as the internal hybridization standard. A secondary antibody (1:2,000; Abcam, Cambridge, United Kingdom) was used for hybridization. A polyvinylidene fluoride (PVDF) membrane (Abcam, Cambridge, United Kingdom) was incubated for 2 min in a 1:1 mixture of enhanced chemiluminescence (ECL) color developer and enhancement solution. Color bands on the membrane were detected with a VersaDoc imaging system (Bio-Rad Laboratories, Hercules, CA, United States).

No Cry1C^^*^ antibody was prepared. Hence, Cry1C^^*^ expression in T1C-19 was detected using an enzyme-linked immunosorbent assay (ELISA), as described by Fu and Liu (2020b). Briefly, ∼20 mg tissue was ground and added to an extraction buffer. The samples were centrifuged at 8,000 × *g* and 4°C for 5 min, the supernatants were diluted, and Cry1C^^*^ expression was detected with a specialized QualiPlate kit (EnviroLogix Inc., Portland, ME, United States). Five samples were pooled per plot and five biological replicates were randomly selected per experiment.

### Antibody Preparation and Hd3a Protein Detection

Mouse anti-Hd3a monoclonal antibodies against Hd3a recombinant protein were prepared by Abmart (Shanghai, China). Briefly, the Hd3a-encoding region with its *his* tag was homologously recombined into a PET-32a vector to transform BL21-DE3. The Hd3a recombinant protein was expressed and purified for mouse inoculation. The immune response was validated by ELISA, and cell fusion, selective culture, hybridoma-positive clone preparation, screening, and large-scale monoclonal antibody generation were performed.

At ∼30 days before flowering, HH1 and MH63 leaf and shoot apex samples were collected and their proteins were extracted with NP-40 lysate (P0013F; Beyotime Biotechnology, Shanghai, China), according to the manufacturer’s instructions. SDS-PAGE and western blot analysis were performed to quantify the Hd3a protein using the aforementioned techniques. The previously prepared monoclonal Hd3a anti-mouse antibody was used at a 1:1,000 dilution for hybridization. The western blot assays were repeated three times.

### Gene Cloning and Expression Vector Construction

The full-length open reading frame (ORF) sequences of the five major flowering genes *Hd1* (AB041838.1), *Hd6* (AB036788.1), *Hd3a* (AB052944.1), *Ehd1* (AB092506.1), *Ghd7* (AB517629.1), and *E3s* (XM_015782466.2) were amplified using MH63 rice cDNA as a template. Gene sequences were procured from the National Center for Biotechnology Information (NCBI; Bethesda, MD, United States) database. The full-length ORF sequences of exogenous *cry1Ab/c* (HQ161055.1) and *cry1C*^^*^ (HM107006) were cloned using HH1 and T1C-19 rice cDNAs as templates.

The primers and recombinant plasmids used in this study are listed in Supplementary Table 1. An In-Fusion HD Cloning Kit Reaction System (TaKaRa Bio Inc., Kusatsu, Shiga, Japan) was established to insert homologous recombinants of *Hd1*, *Hd6*, *Hd3a*, *Ehd1*, and *Ghd7* into the subcellular localization vector pBINPLUS-GFP (GFP), the BiFC vector PCV-cYFP-N (nYFP), and the yeast two-hybrid vector pGBKT7 (BD) *via* a single BamH digestion site. Exogenous *cry1Ab/c* was cloned into the subcellular location vector pBINPLUS-mCherry (mCherry), the BiFC vector PCV-nYFP-C (cYFP), and the yeast two-hybrid vector pGADT7 (AD). Exogenous *cry1C*^∗^ expressed by transgenic T1C-19 was cloned into the BiFC vector cYFP.

### Protoplast Isolation and Rice Transfection

Rice protoplasts were isolated and transfected according to a previously published method (Zhang et al., 2011). Protoplasts with a diameter of ∼7–25 μm were generated, incubated for 16–20 h at room temperature (20–25°C), and visualized at 63× under the oil mirror lens of a confocal laser scanning microscope (ZEISS LSM710nlo; Carl Zeiss AG, Oberkochen, Germany). GFP, YFP, and mCherry were excited at 488, 514, and 561 nm, respectively. All fluorescence experiments were independently repeated at least thrice.

### Transient Expression System Developed *via Agrobacterium*-Mediated Transformation

A transient expression system was developed *via Agrobacterium*-mediated transformation according to a previously published method (Zhang et al., 2011). *N. benthamiana* leaves were perforated, excised, and mounted with water on microscope slides. Green GFP, red mCherry, and yellow YFP fluorescence were observed under a confocal laser scanning microscope (ZEISS LSM710nlo; Carl Zeiss AG, Oberkochen, Germany).

### Yeast Two-Hybrid Assay

A yeast two-hybrid point-to-point verification system based on the Matchmaker Gold yeast two-hybrid system was constructed (Clontech Laboratories, Mountain View, CA, United States). The target genes infused to the AD and BD vectors were transformed into competent Y2H Gold yeast cells. The co-transformed yeast cells were coated onto SD-Trp-Leu-His-Ade medium with X-α-gal for 4 days.

### Bimolecular Fluorescence Complementation Assay

The target genes infused to the nYFP and cYFP vectors were transformed into *Agrobacterium* sp. strain GV3101, which, in turn, infiltrated *N. benthamiana* leaves (Zhang et al., 2011). The 4A-nYFP (NbeIF4A) and P2-cYFP (RSV-encoded proteins) served as positive controls. After 48 h, YFP fluorescence signals were observed at 514 nm under a Zeiss LSM750 confocal laser scanning microscope (Carl Zeiss AG, Oberkochen, Germany).

### Co-immunoprecipitation Assay

Co-immunoprecipitation was performed as described previously (Fu and Liu, 2020a). Briefly, *N. benthamiana* leaves that had been co-transformed with the target genes were pulverized, and their protein content was extracted. GFP-trap beads were used to immunoprecipitate the protein extracts and then washed 6× with immunoprecipitation buffer. The agarose beads were diluted with 5× sodium dodecyl sulfate (SDS) buffer and boiled at 95°C for 10 min for subsequent use in SDS-PAGE and western blot analysis.

### Detection of Hd3a Protein Expression Levels

Proteins derived from co-transformed Hd3a-GFP4, Cry1Ab/c-mCherry, and E3s-mCherry and from co-transformed Hd3a-GFP and Cry1Ab/c-mCherry in *N. benthamiana* leaves were extracted after co-expression for 48 h, according to the aforementioned method. The proteins were quantitated using the bicinchoninic acid (BCA) (Beyotime Biotechnology, Shanghai, China) method and western blot analysis. The average gray level of the western blot strip was detected with ImageJ software (NIH, Bethesda, MD, United States). β-Actin was used as the internal quantitative control. Western blot assays were repeated three times.

### Data Analysis

All the data analyses were performed using SPSS 20.0 statistical software. With regard to flowering time, the expression of some genes and western blot band density were analyzed, and independent *t*-tests were developed to identify the significant differences among different treatments.

## Results

### Flowering Times of Transgenic Rice HH1 and T1C-19 and Parental Rice MH63

**FIGURE 1:**
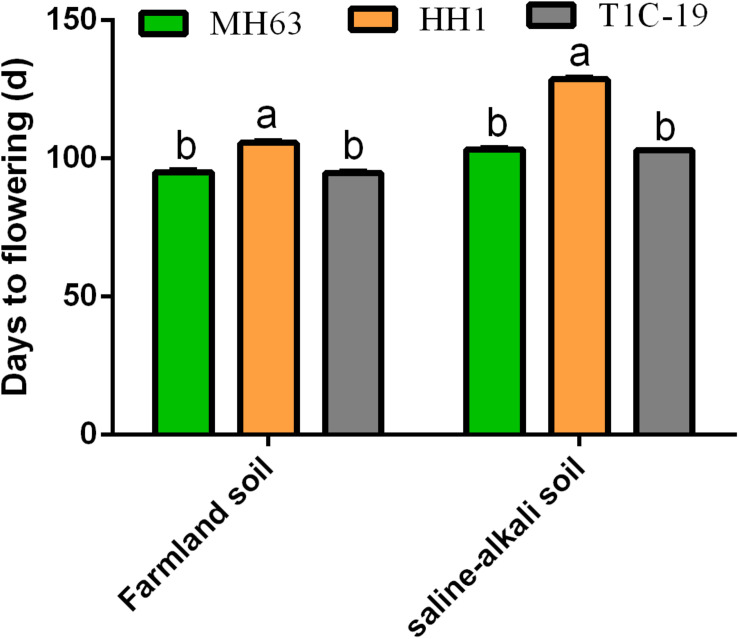
Flowering times of insect-resistant transgenic rice HH1 and TIC-19 and parental rice MH63 grown on farmland and saline–alkali soils (*n* = 50). The flowering time was recorded as the time of emergence of the first panicle in a single plant, and 50 rice plants per treatment were randomly selected to record the total days to flowering. Lowercase letters indicate significant differences among treatment means (*P* ≤ 0.05; Student’s *t*-test).

To determine whether the flowering time of transgenic rice plants was regulated by foreign genes alone, we designed two different environment conditions (farmland or saline–alkaline soils) to measure the total days to flowering. On farmland soil, MH63 and T1C-19 flowered at 95 days, whereas HH1 flowered at 107 days. On saline–alkaline soil, MH63 and T1C-19 flowered at 103 days, whereas HH1 flowered at 128 days. Therefore, the flowering time for HH1 was delayed by 12 and 25 days on farmland and saline–alkaline soils, respectively. Furthermore, subsequent growth stages were also delayed (Figure 1 and Supplementary Figure 1).

### Transcriptional and Translational Detection of Foreign Genes in Transgenic Rice Plants

**FIGURE 2:**
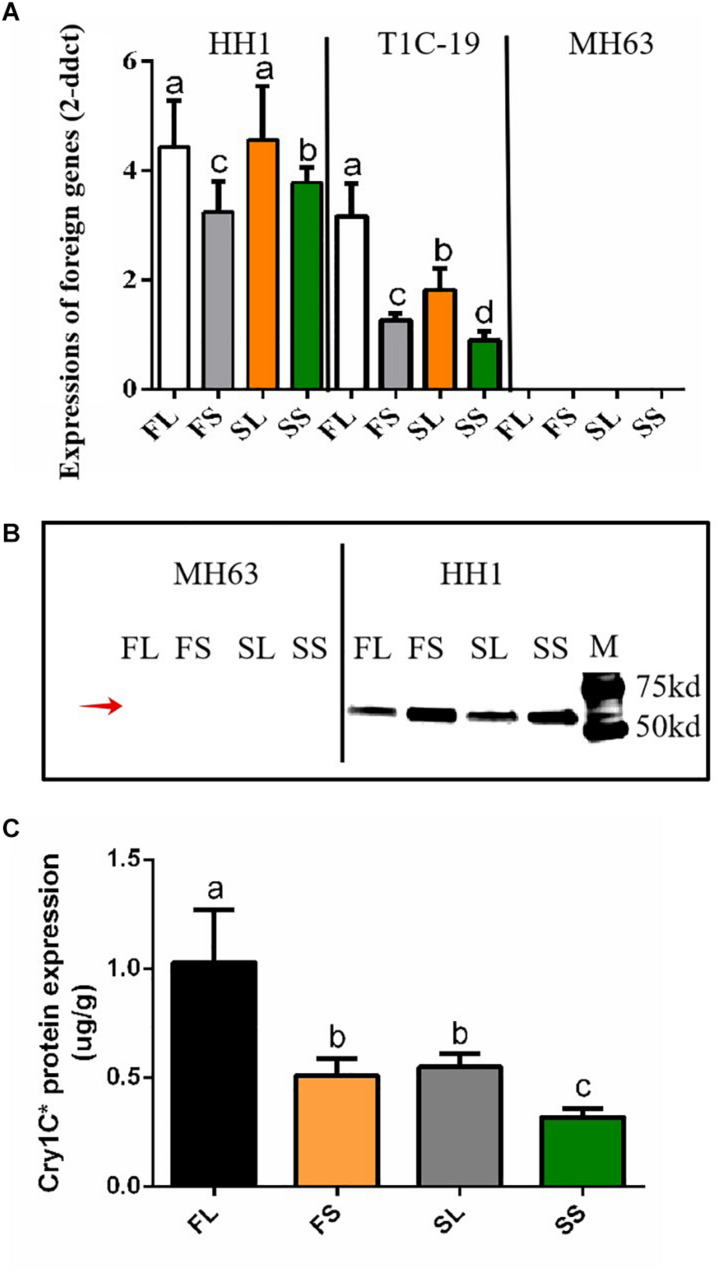
The detection of transcription and translation of foreign genes in transgenic rice HH1 and T1C-19 and parental rice MH63. **(A)** The expression of foreign *cry1Ab/c* and *cry1C*^^*^ expressed by HH1 or T1C-19 in the leaves (L) and shoot apices (S) grown in farmland (F) or saline–alkali (S) soils, according to the RT-qPCR method (*n* = 5; *P* ≤ 0.05, Student’s *t*-test). **(B)** The expression of Cry1Ab/c (67 kDa) protein in the leaves (L) and shoot apices (S) of HH1 grown in farmland (F) or saline–alkali (S) soils, as quantified by western blotting. The red arrow indicates the target protein band. The protein expression assay was repeated three times. **(C)** Cry1C^^*^ protein expression in the leaves (L) and shoot apices (S) of T1C-19 grown in farmland (F) or saline–alkali (S) soils, as evaluated by the enzyme-linked immunosorbent assay (*n* = 5; *P* ≤ 0.05, Student’s *t*-test). (FL) leaves of rice grown in farmland soil; (FS) shoot apices of rice grown in farmland soil; (SL) leaves of rice grown in saline–alkali soil; (SS) shoot apices of rice grown in saline–alkali soil.

To determine whether the foreign genes are normally transcribed and expressed in HH1 and T1C-19, we collected leaf samples and detected these parameters *via* qRT-PCR and western blotting or ELISA grown on farmland and saline–alkaline soils during the heading stage. Results showed that, for the farmland and saline–alkaline soil treatments, *cry1Ab/c* and *cry1C*^^*^ were transcribed and expressed in transgenic rice lines HH1 and T1C-19, respectively, but not in parental MH63 (Figure 2).

### Hd3a Expression Levels Were Altered in HH1

**FIGURE 3:**
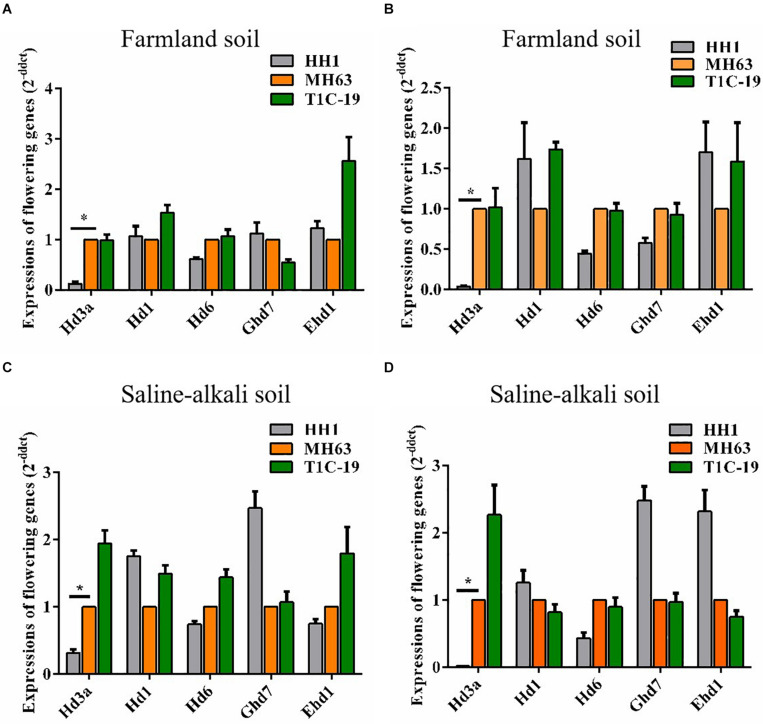
Relative expression of *Hd3a*, *Hd1*, *Hd6*, *Ghd7*, and *Ehd1* in the leaves and shoot apices of insect-resistant transgenic rice HH1 and T1C-19 and parental rice MH63 grown on farmland and saline–alkali soils (*n* = 5). At ∼30 days before flowering, the leaf and shoot apex samples were collected from HH1, T1C-19, and MH63 for detecting *Hd3a* expression: **(A,C)** leaf; **(B,D)** shoot apex; * indicates significant differences among treatment means (*P* ≤ 0.05; Student’s *t*-test).

Compared to parental MH63 rice, Flowering was delayed in transgenic HH1. Therefore, we investigated whether the relative transcription levels of key flowering genes were altered in HH1. At ∼30 days before flowering, we collected leaf and shoot apex samples and measured the relative expression levels of *Hd3a*, *Hd1*, *Hd6*, *Ghd7*, and *Ehd1* in HH1, T1C-19, and MH63 grown on farmland and saline–alkaline soils during flowering time. The *Hd3a* expression levels in the leaves and shoot apices of HH1 grown on farmland were 7× and 25× lower, respectively, than in those of MH63. The *Hd3a* expression levels in the leaves and shoot apices of HH1 grown on saline–alkaline soil were 3× and 50× lower, respectively, than in those of MH63. Moreover, all differences were significant. However, the transcriptional levels of *Hd1*, *Hd6*, *Ghd7*, and *Ehd1* only slightly and non-significantly differed between HH1 and MH63 under both soil conditions (Figure 3). The transcription levels of all five major flowering genes only slightly and non-significantly differed between T1C-19 and MH63 under both soil conditions. Thus, *Hd3a* downregulation may be associated with delayed flowering in transgenic HH1 rice (Figure 3).

**FIGURE 4:**
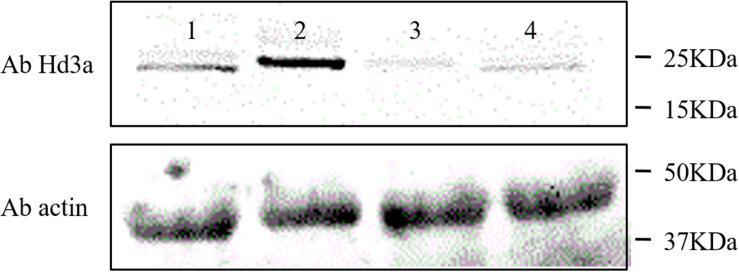
Western blot quantification of Hd3a (∼25 kDa) expression levels in the leaf and shoot apex of insect-resistant transgenic rice HH1 and parental rice MH63. At ∼30 days before flowering, HH1 and MH63 leaf and shoot apex samples were collected and their proteins were extracted, to analyze Hd3a expression. (Lane 1) leaf in MH63. (Lane 2) shoot apex in MH63. (Lane 3) leaf in HH1. (Lane 4) shoot apex in HH1. Loading quantity was determined to be 20 μg by the BCA method. β-Actin was used as the internal normalization control. The western blot assays were repeated three times.

We also detected the *Hd3a* expression levels in the leaves and shoot apices of HH1 and MH63 grown on farmland soil at the same stage. Western blotting revealed that the Hd3a expression levels in HH1 leaves and shoot apices were significantly lower than in those of MH63. This finding was consistent with the fact that *Hd3a* transcription levels in the same tissues were significantly lower in HH1 than those in MH63 raised under the same growth conditions. Therefore, the florigen Hd3a may regulate the flowering progress in transgenic HH1 rice (Figure 4).

### Exogenous Cry1Ab/c Protein Interacted With Hd3a

**FIGURE 5:**
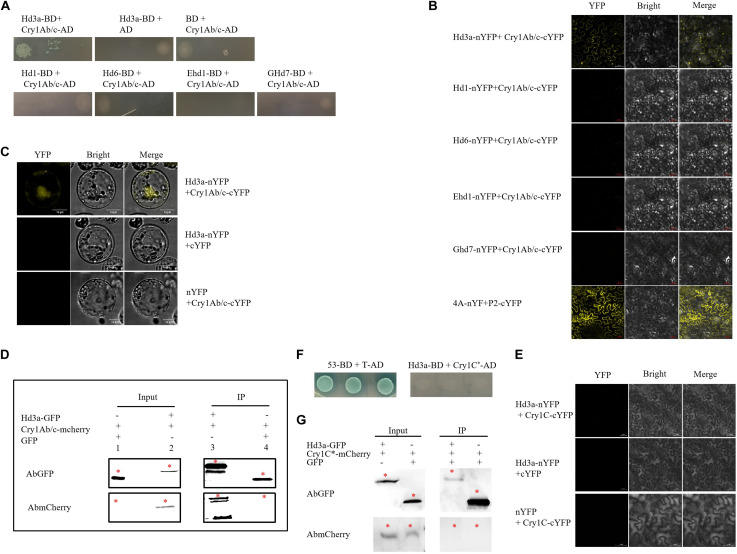
Interaction between exogenous Cry1Ab/c protein and Hd3a. **(A)** The interactions among major rice flowering proteins, i.e., Hd3a, Hd1, Hd6, Ehd1, and Ghd7 and Cry1Ab/c in Y2H Gold; yeast strain Y2H Gold co-transformed with indicated plasmids and spotted on quadruple dropout media with X-α-gal. Empty vectors pGBKT7 (BD) and pGADT7 (AD) were used as negative controls. The yeast two-hybrid assay was repeated three times. **(B)** Bimolecular fluorescence complementation (BiFC) analysis of interactions among Hd3a, Hd1, Hd6, Ehd1, and Ghd7 and Cry1Ab/c in tobacco mesophyll cells, as indicated by YFP yellow fluorescence; 4A (NbeIF4A) plus P2 (RSV-encoded proteins) was used as the positive control. Scale bars: 50 μm. The BIFC assay was repeated three times. **(C)** BiFC analysis of interactions between Cry1Ab/c and Hd3a in rice protoplasts. Scale bars: 10 μm. **(D)** Co-IP analysis of interactions between Hd3a-GFP and Cry1Ab/c-mCherry fusion proteins. Protein extracts (input) were immunoprecipitated with GFP-trap beads (IP) and resolved by SDS-PAGE. Immunoblots were developed with the GFP antibody (AbGFP) to detect Hd3a-GFP (45 kDa) and were developed with the mCherry antibody (AbmCherry) to detect the Cry1Ab/c-mCherry protein (94 kDa). GFP empty vector plus Cry1Ab/c-mCherry was used as the negative control. * indicates the target band. The co-IP assay was repeated three times. **(E)** BiFC analysis of interactions between Cry1C^∗^ expressed by insect-resistant transgenic rice T1C-19 and Hd3a in tobacco mesophyll cells. Scale bars: 50 μm. The BIFC assay was repeated three times. **(F)** The interactions between Hd3a and Cry1C^∗^ in Y2H Gold; yeast strain Y2H Gold co-transformed with indicated plasmids and spotted on quadruple dropout media with X-α-gal. Yeast strain Y2H Gold was co-transformed with vectors 53-BD and T7-AD and it was used as the positive control. **(G)** Co-IP analysis of interactions between Hd3a-GFP and Cry1C^∗^-mCherry fusion proteins. Protein extracts (input) were immunoprecipitated with GFP-trap beads (IP) and resolved by SDS-PAGE. Immunoblots were developed with the GFP antibody (AbGFP) to detect Hd3a-GFP (45 kDa) and with mCherry antibody (AbmCherry) to detect the Cry1C^∗^-mCherry protein (96 kDa). GFP empty vector plus Cry1C^∗^-mCherry was used as the negative control. * indicates the target band. The co-IP assay was repeated three times.

We used yeast two-hybrid assays to explore possible interactions among the flowering regulators Hd3a, Hd1, Hd6, Ghd7, and Ehd1 and explain delayed flowering in HH1. The Hd3a-BD and Cry1Ab/c-AD yeast co-transformants grew on a quadruple dropout medium containing X-α-gal. However, the Hd1-BD, Hd6-BD, Ghd7-BD, Ehd1-BD, and Cry1Ab/c-AD yeast co-transformants did not thrive on the aforementioned medium (Figure 5A). Results of the BiFC assays revealed that only co-transformants comprising Hd3a were fused to the *N*-terminal of YFP and Cry1Ab/c fused to the *C*-terminal of YFP demonstrated strong fluorescence signals in their nuclei and cytosols. Thus, physical interactions occurred between Hd3a and Cry1Ab/c in the tobacco mesophyll cells. Nevertheless, the co-transformation between the flowering proteins Hd1, Hd6, Ghd7, Ehd1, and Cry1Ab/c did not result in strong YFP fluorescence (Figure 5B). Hd3a fused to the *N*-terminal of YFP and Cry1Ab/c fused to the *C*-terminal of YFP displayed strong fluorescence signals in rice protoplasts (Figure 5C). Co-IP assays further showed that Cry1Ab/c-mCherry fusion proteins could be immunized down with Hd3a-GFP fusion proteins, whereas GFP could not (Figure 5D). However, the co-expression of Hd3a, Hd1, Hd6, Ghd7, Ehd1, and exogenous Cry1C^^*^ expressed by transgenic T1C-19 rice did not exhibit YFP yellow fluorescence in tobacco mesophyll cells (Figure 5E and Supplementary Figure 3). Further, the Hd3a-BD and Cry1C^^*^-AD yeast co-transformants did not grow on the quadruple dropout medium with X-α-gal (Figure 5F). Consistently, the co-IP assays showed that Cry1C^^*^-mCherry fusion protein could not be immunized down with Hd3a-GFP fusion protein (Figure 5G). These data support the theory that Hd3a physically interacts with Cry1Ab/c in the cytoplasm and nuclei, while Cry1C^^*^ could not interact with it. Subcellular localization analysis using confocal laser scanning microscopy corroborated the results of BiFC analyses and indicated that Hd3a and Cry1Ab/c were co-localized to the nuclei and cytosol (Supplementary Figure 2).

### Interaction Between Hd3a and Cry1Ab/c Protein Inhibited Hd3a Expression by Mediating the E3s Ubiquitin Proteasome Pathway

**FIGURE 6:**
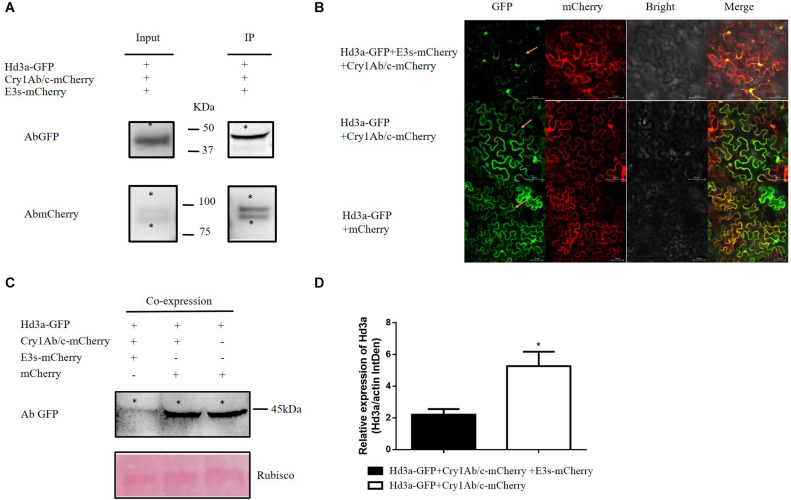
Co-expression of Hd3a-GFP4, Cry1Ab/c-mCherry, and E3s-mCherry inhibits Hd3a protein expression in tobacco mesophyll cells. **(A)** Co-IP analysis of interactions between Hd3a-GFP4, Cry1Ab/c-mCherry, and E3s-mCherry. Protein extracts (input) were immunoprecipitated with GFP-trap beads (IP) and resolved by SDS-PAGE. Immunoblots were developed with the GFP antibody (AbGFP) to detect Hd3a-GFP (45 kDa) and with mCherry antibody (AbmCherry) to detect Cry1Ab/c-mCherry (94 kDa) and E3s-mCherry (87 kDa) fusion proteins. * indicates the target band. The co-IP assay was repeated three times. **(B)** The fluorescence intensity of Hd3a-GFP was detected in tobacco mesophyll cells co-expressing Hd3a-GFP4, Cry1Ab/c-mCherry, and E3s-mCherry using a laser confocal microscope. The co-expression of Hd3a-GFP and Cry1Ab/c-mCherry, Hd3a-GFP and mCherry were used as the controls. **(C)** The expression of Hd3a proteins and co-expression of Hd3a-GFP4, Cry1Ab/c-mCherry, and E3s-mCherry was detected quantitatively (20 μg) by western blotting; co-expressed Hd3a-GFP and Cry1Ab/c-mCherry was used as the control; Rubisco (bottom) was used as loading sample control. **(D)** Western blot band density of Hd3a protein expression (relative to actin) was analyzed using ImageJ software; * indicates significant differences between treatment means (*P* ≤ 0.05; Student’s *t*-test). The western blot assays were repeated three times.

Because Hd3a interacts with exogenous Cry1Ab/c, we postulated that this interaction regulates flowering time by modulating Hd3a protein expression. We detected this mechanism *via* the transient co-expression of Hd3a-GFP and Cry1Ab/c-mCherry in tobacco mesophyll cells. Hd3a protein expression did not significantly differ between co-expressed Hd3a-GFP and Cry1Ab/c-mCherry and between co-expressed Hd3a-GFP and mCherry (Figure 6A). Consequently, another protein might participate in this process.

The E3 ubiquitin proteasome pathway is vital for protein degradation in eukaryotes. In this study, we detected interactions between Hd3a-GFP4, Cry1Ab/c-mCherry, and E3s-mCherry. Hd3a-GFP pulled down Cry1Ab/c-mCherry and E3s-mCherry. Therefore, interactions occurred among all three proteins (Figure 6B). We used western blotting to establish whether the Cry1Ab/c-mediated decrease in Hd3a protein expression depended on the E3 ubiquitin ligase pathway. To this end, we first transiently co-expressed Hd3a-GFP, Cry1Ab/c-mCherry, and E3s-mCherry in tobacco mesophyll cells. The Hd3a expression levels in co-expressing Hd3a-GFP, Cry1Ab/c-mCherry, and E3s-mCherry tobacco mesophyll cells were significantly lower than those in co-expressing Hd3a-GFP and Cry1Ab/c-mCherry and co-expressing Hd3a-GFP and mCherry (Figures 6C,D) cells. Therefore, we speculated that when the Cry1Ab/c protein interacted with Hd3a protein, it inhibited the expression of the latter by mediating the E3s ubiquitin proteasome pathway. The net effect is delayed flowering in transgenic HH1 rice, compared with that in parental MH63 rice.

## Discussion

In the present study, we showed that the flowering time of insect-resistant transgenic *cry1Ab/c* rice line HH1 was delayed, compared with that of the parental rice line MH63, cultivated in farmland or saline–alkaline soils. In contrast, the flowering times of the insect-resistant transgenic *cry1C*^^*^ rice lines T1C-19 and MH63 remained the same. Therefore, T1C-19, which would not affect the flowering time, is a good control for HH1. In addition, the flowering time of HH1 was more greatly delayed than that of parental MH63 under saline–alkali conditions, compared with that under farmland conditions. It is evident that flowering time is regulated by many factors; it may be regulated not only by environmental factors but also by exogenous genes. *Hd3a* expression was significantly lower in HH1 than in MH63, but was not significantly lower in T1C-19, and the Cry1Ab/c expressed by HH1 and not the foreign Cry1C^^*^ expressed by T1C-19 interacted with florigen Hd3a *in vitro* and *in vivo* and inactivated the latter *via* the E3 ubiquitin ligase pathway. Thus, the expression of Cry1Ab/c might have mediated the observed reduction in Hd3a expression through the E3 ubiquitin ligase pathway. Hence, we assumed that the decrease in *Hd3a* expression and the interaction between Cry1Ab/c and Hd3a might cooperatively regulate flowering progress in transgenic HH1 rice. Taken together, two transgenic rice plants with identical genetic backgrounds had different flowering times under the same growth conditions. It is evident that not all foreign genes introduced into rice would cause a flowering delay, and the interaction between foreign proteins and flowering proteins occurs as an individual case. The delayed flowering of HH1 rice could be attributable to the interaction between the foreign protein Cry1Ab/c and flowering protein Hd3a.

The insertion of foreign genes into rice genomes might cause morphophysiological variations, including the alteration of the flowering time. It is reported that the flowering times of transgenic rice plants containing toxin *cry1Ab*, neomycin phosphotransferase II (*nptII*), *cry1Ac/epsps*, or *bar/pin* were delayed, compared with that of parental rice, and this decreased the fertility of the former (Schuh et al., 1993; Lynch et al., 1995; Cui et al., 1998; Cheng, 2002; Wang, 2015). Xia et al. (2011) found that the transgenic rice HH1 showed slightly lower values of 1,000-grain weight than its parental MH63 under environmental conditions with low insect pressure, which may be related to the delay in the growth stage of HH1. Similarly, our previous results showed that the flowering time (unpublished data) and yield-related traits, as measured by filled grain number and filled grain weight per plant, were significantly lower for HH1 than for the parental rice strain MH63 grown under farmland or saline–alkaline soil conditions without target insect pressure (Fu et al., 2018). Therefore, we predicted that the Cry1Ab/c protein expressed by HH1 decreased the rice yield by affecting its flowering time. The current study is a continuation of our previous study. In the long term, the flowering time of transgenic *cry1Ab/c* HH1 and not that of *cry1C*^^*^ T1C-19 rice was more greatly delayed than that of MH63. Hence, we assumed that there may be no gene flow between HH1 and MH63. Furthermore, large-scale HH1 applications could be ecologically safe and beneficial despite the morphophysiological differences between this cultivar and MH63.

Additionally, an analysis of transgenic rice expressing GFP-tagged Hd3a showed that Hd3a is synthesized in the leaf vascular tissue and transported *via* the phloem toward the shoot tip meristem, which Hd3a transforms into an inflorescence meristem (Tamaki et al., 2007). Hd3a overexpression induces an early-flowering rice phenotype, whereas Hd3a knockdown *via* RNA interference (RNAi) delays rice flowering. Therefore, H3da expression is positively correlated with flowering time (Kojima et al., 2002; Tamaki et al., 2007; Komiya et al., 2008). In the present study, the Hd3a transcription and translation levels in HH1 leaves and shoot apical meristems were significantly lower than those in MH63 grown on farmland and saline–alkaline soils. In contrast, the Hd3a translation levels in T1C-19 leaves and shoot apical meristems did not significantly differ from those in MH63 grown on farmland and saline–alkaline soils. Hence, we propose that the exogenous *cry1Ab/c* expressed in HH1 rice regulates flowering time, by controlling Hd3a expression.

Various endogenous rice proteins such as GF14c (14-3-3), OsKANADI, and BIP116b interact with Hd3a and modulate signal transduction for the flowering process (Purwestri et al., 2017). Taoka et al. (2011) found that Hd3a interacts with the GF14c (14-3-3) protein in shoot apical cells and forms a complex that is translocated to the nucleus, where it binds the *Oryza sativa* FD1 transcription factor. Thus, the induction of *OsMADS15* transcription promotes flowering. The foreign Cry1Ab/c protein expressed by HH1 strongly interacts with Hd3a, inhibits Hd3a protein expression, and delays flowering. Rice varieties exhibiting GF14c knockout and transgenic overexpression revealed that the GF14c (14-3-3) protein interacts with Hd3a florigen and negatively regulates rice flowering (Purwestri et al., 2009). Interactions among endogenous or exogenous proteins and Hd3a might regulate rice flowering.

Several RING finger proteins such as E3 ubiquitin ligases control flowering, phytohormone signaling, and stress response in plants (Du et al., 2016; Wu et al., 2016). In *Arabidopsis*, ubiquitin-mediated protein degradation controls the expression and stability of flowering regulators. Most ubiquitination marker proteins are degraded by the 26S proteasome (Sarid-Krebs et al., 2015; Yang et al., 2015). Here, foreign Cry1Ab/c and Hd3a strongly interacted with E3 ubiquitin ligases. Cry1Ab/c-mCherry, Hd3a-GFP, and E3-mCherry co-expression and not Cry1Ab/c-mCherry or Hd3a-GFP co-expression significantly downregulated Hd3a. Hence, the observed Cry1Ab/c-mediated decrease in Hd3a protein expression might depend on the E3 ubiquitin ligase pathway. We propose that Cry1Ab/c expression regulates rice flowering time by ubiquitinating the florigen Hd3a. Nevertheless, additional experimentation is required to confirm this hypothesis.

To the best of our knowledge, this study is the first to elucidate the molecular mechanisms underlying the unintended effects of protein interactions in transgenic crops and identify Hd3a as an endogenous target protein of foreign Cry1Ab/c. The insertion of foreign *cry1Ab/c* into rice genomes and its transcription produced the Cry1Ab/c protein that could regulate flowering time by downregulating the expression of the Hd3a gene and protein. These discoveries provided an empirical foundation for clarifying the positive and negative effects of transgenic plant production, prediction of their potential risks, and the design of innovative strategies for breeding insect resistance in crops. Further research is required to determine how Cry1Ab/c affects *Hd3a* expression and cooperatively regulates the flowering signal with Hd3a protein in HH1 transgenic rice, and to rule out the possibility that the endogenous flowering time gene was destroyed by the T-DNA insertion event.

## Data Availability Statement

The original contributions presented in the study are included in the article/Supplementary Material, further inquiries can be directed to the corresponding author/s.

## Author Contributions

JF designed and executed the experiments and wrote the manuscript. BL designed and helped revise the manuscript. GL helped revise the manuscript. All authors contributed to the article and approved the submitted version.

## Conflict of Interest

The authors declare that the research was conducted in the absence of any commercial or financial relationships that could be construed as a potential conflict of interest.
