# Supplementary material for: Foreign Cry1Ab/c Delays Flowering in Insect-Resistant Transgenic Rice via Interaction With Hd3a Florigen
Source: Front Plant Sci. 2021 Feb 11;12:608721. doi: 10.3389/fpls.2021.608721 (PMC7905309; doi:10.3389/fpls.2021.608721)
Supplement: Supplementary file 1 [file Data_Sheet_1.docx]

**Supplementary material**


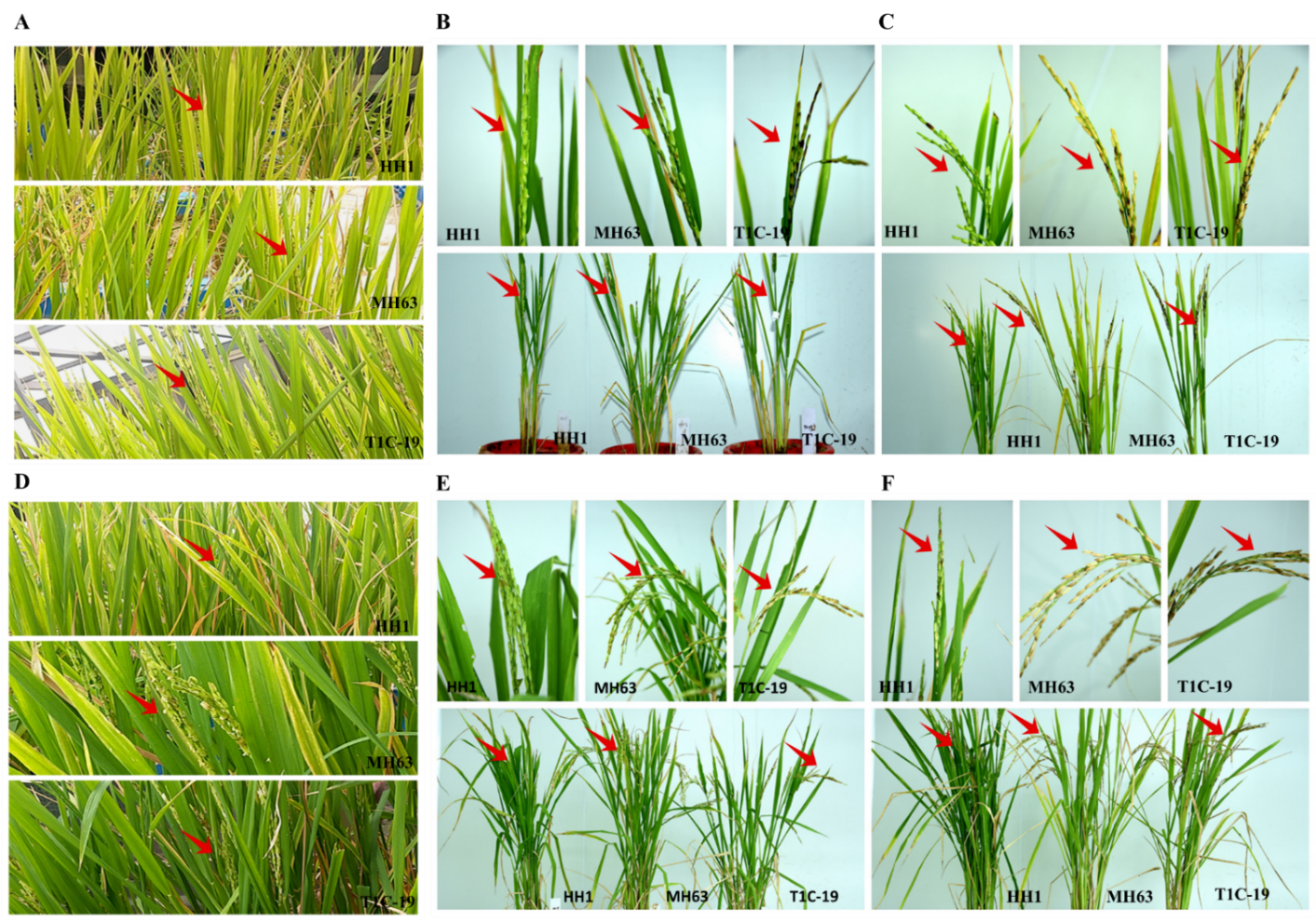


Fig. S1 Flowering time difference between insect-resistant transgenic rice HH1、TIC-19 and parental rice MH63 during three reproductive stages in saline-alkali ( A, B , C) and farmland (D, E, F) soils. (A,D) Heading stage, (B, E) Filling stage, (C, F) Maturing stage, The arrowhead points to the spike of rice.


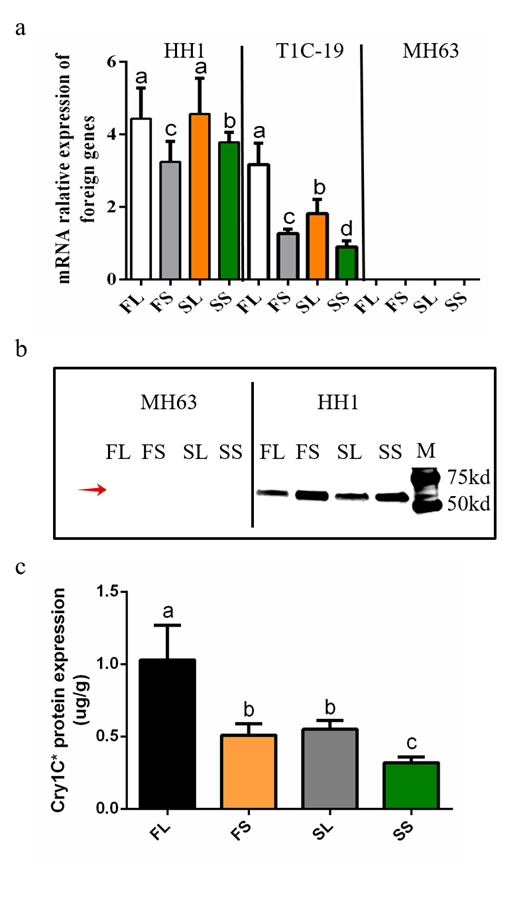


Fig. S2 The cry1Ab/c transcription and translation in transgenic rice HH1, T1C-19, and parental rice MH63. A, relative cry1Ab/c and cry1C* mRNA expression levels in leaves (L) and shoot apices (S) of HH1 and T1C-19 grown in saline-alkali (S) and farmland (F) soils according to RT-Qpcr method. B, Cry1Ab/c (67 kDa) protein expression in leaves (L) and shoot apices (S) of HH1 and T1C-19 grown in saline-alkali and farmland soils as quantified by western blot. Red arrow indicates target protein band. C, Cry1C* protein expression in leaves (L) and shoot apices (S) of HH1 and T1C-19 grown in saline-alkali and farmland soils as evaluated by enzyme-linked immunosorbent assay. (FL) leaves of rice grown in farmland soil; (FS) shoot apices of rice grown in farmland soil; (SL) leaves of rice grown in saline-alkali soil; (SS) shoot apices of rice grown in saline-alkali soil.


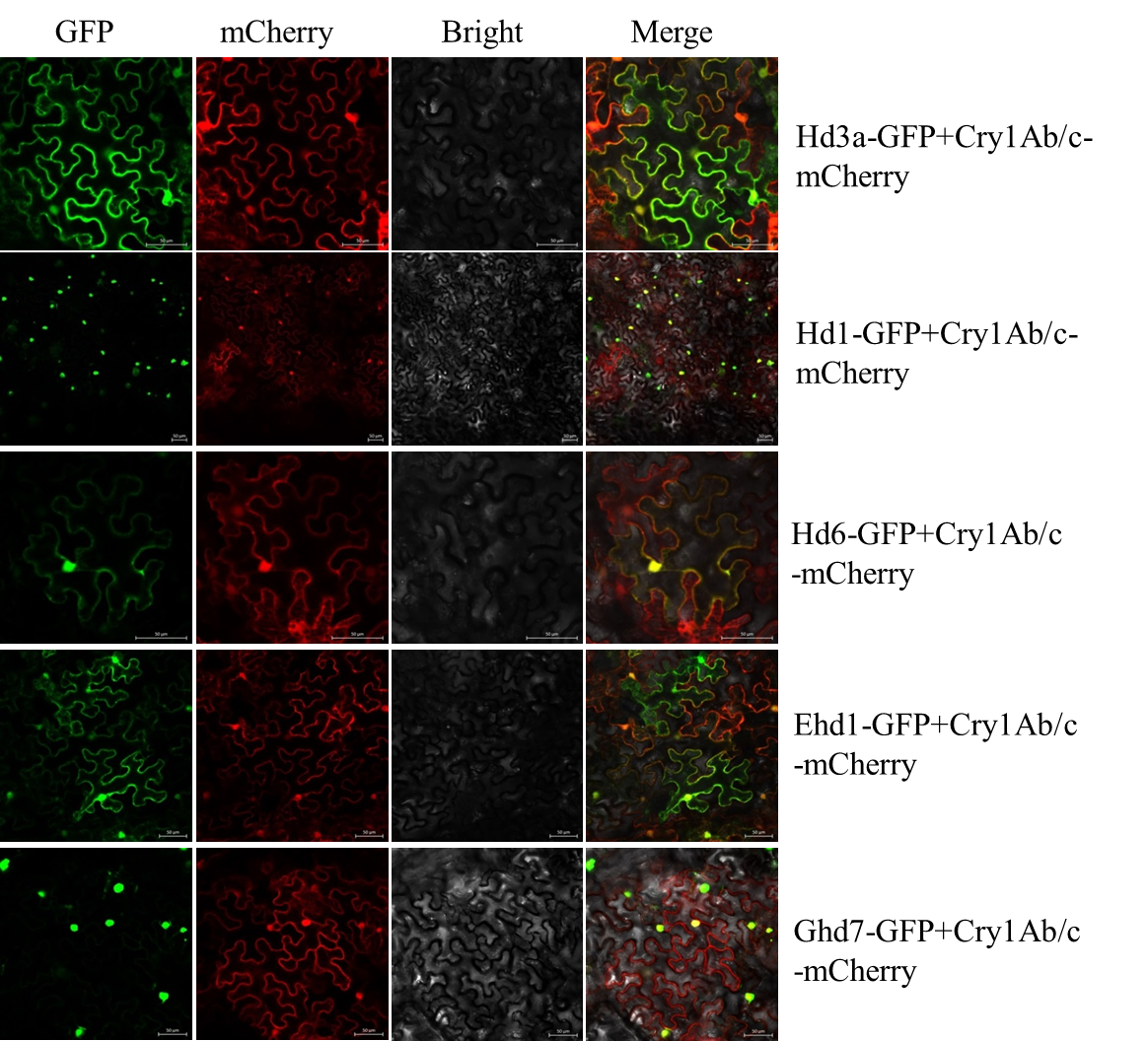


Fig. S3 The subcellular co-localization of full-length of Ghd7, Ehd1, Hd6, Hd1, Hd3a in tobacco mesophyll cells. Flowering genes were fused with GFP and cry1Ab/c gene was fused with mCherry at their C terminal under control of the 35S promoter (Scale bars: 50μm).

**Table S1 Primers used in this study.**

| **Primer** | **Sequences (5’-3’)** |
| --- | --- |
| **Subcellular localization** |  |
| Hd3a-GFP-F | CGGGGTCGACGGATCCATGGCCGGAAGTGGCAGG |
| Hd3a-GFP-R | TGCTCACCATGGATCCGGGGTAGACCCTCCTGCC |
| Hd1-GFP-F | CGGGGTCGACGGATCCATGAATTATAATTTTGGTGGCAA |
| Hd1-GFP-R | TGCTCACCATGGATCCGAACCATGGAACAGTACCATAG |
| Hd6-GFP-F | CGGGGTCGACGGATCCATGACCGATGCGCCTCC |
| Hd6-GFP-T | TGCTCACCATGGATCCTTGTGGTCGTGCTCTGCT |
| Ghd7-GFP-F | CGGGGTCGACGGATCCATGTCGATGGGACCAGCAG |
| Ghd7-GFP-R | TGCTCACCATGGATCCTCTGAACCATTGTCCAAGCTC |
| Ehd1-GFP-F | CGGGGTCGACGGATCCATGGATCACCGAGAGCTGTGG |
| Ehd1-GFP-R | TGCTCACCATGGATCCGAAATTCCAAAAACATGGTCCA |
| Cry1Ab/c-mCherry-F | CGGGGTCGACGGATCCATGGACAACTGCAGGCCATAC |
| Cry1Ab/C-mCherry-R | TGCTCACCATGGATCCTTCAGCCTCGAGTGTTGC |
| E3s-mCherry-F | CGGGGTCGACGGATCCATGGACAAATCAGAAGAGGATGC |
| E3s-mCherry-R | TGCTCACCATGGATCCTGTGGTTTTTCCAATGCCT |
| **BIFC** |  |
| Hd3a-nYFP-F | CGGGAGATGCGGATCCATGGCCGGAAGTGGCAGG |
| Hd3a-nYFP-R | GCTCGCCTGGGGATCCCTAGGGGTAGACCCTCCTGCC |
| Hd1-nYFP-F | CGGGAGATGCGGATCCATGAATTATAATTTTGGTGGCAA |
| Hd1-nYFP-R | GCTCGCCTGGGGATCCTCAGAACCATGGAACAGTACCAT |
| Hd6-nYFP-F | CGGGAGATGCGGATCCATGACCGATGCGCCTCC |
| Hd6-nYFP-R | GCTCGCCTGGGGATCCTCATTGTGGTCGTGCTCTGCT |
| Ghd7-nYFP-F | CGGGAGATGCGGATCCATGTCGATGGGACCAGCAG |
| Ghd7-nYFP-R | GCTCGCCTGGGGATCCCTATCTGAACCATTGTCCAAGCTC |
| Ehd1-nYFP-F | GGGAGATGCGGATCCATGGATCACCGAGAGCTGTGG |
| Ehd1-nYFP-R | GCTCGCCTGGGGATCCCTAGAAATTCCAAAAACATGGTC |
| Cry1Ab/c-cYFP-F | CGGGAGATGCGGATCCATGGACAACTGCAGGCCATAC |
| Cry1Ab/c-cYFP-R | GCTCGCCTGGGGATCCTTATTCAGCCTCGAGTGTTGC |
| Cry1C^*^-cYFP-F | CGGGAGATGCGGATCCATGGAGGAGAACAATCAGAACC |
| Cry1C^*^-cYFP-R | GCTCGCCTGGGGATCCCTACTTTTGTGCTCTTTCAAGGTC |
| **Yeast two-hybrid** |  |
| Hd3a-BD-F | CATGGAGGCCGAATTCATGGCCGGAAGTGGCAGG |
| Hd3a-BD-R | GGATCCCCGGGAATTCTAGGGGTAGACCCTCCTGCC |
| Hd1-BD-F | CATGGAGGCCGAATTCATGAATTATAATTTTGGTGGCAA |
| Hd1-BD-R | GGATCCCCGGGAATTCTCAGAACCATGGAACAGTACCAT |
| Hd6-BD-F | CATGGAGGCCGAATTCATGACCGATGCGCCTCC |
| Hd6-BD-R | GGATCCCCGGGAATTCTCATTGTGGTCGTGCTCTGCT |
| Ghd7-BD-F | CATGGAGGCCGAATTCATGTCGATGGGACCAGCAG |
| Ghd7-BD-R | GGATCCCCGGGAATTCCTATCTGAACCATTGTCCAAGCT |
| Ehd1-BD-F | CATGGAGGCCGAATTCATGGATCACCGAGAGCTGTGG |
| Ehd1-BD-R | GGATCCCCGGGAATTCCTAGAAATTCCAAAAACATGGTC |
| Cry1Ab/c-AD-F | GGAGGCCAGTGAATTCATGGACAACTGCAGGCCATAC |
| Cry1Ab/c-AD-R | CACCCGGGTGGAATTCTTATTCAGCCTCGAGTGTTGC |
| **RT-qPCR** |  |
| Cry1Ab/c-qF | GAAGGTTTGAGCAATCTCTAC |
| Cry1Ab/c-qR | CGATCAGCCTAGTAAGGTCGT |
| Cry1C^*^-qF | ATCACAGAGGTTCCTCCCCA |
| Cry1C^*^-qR | TGCCACCTCGTGAAGGATAC |
| Hd3a-qF | GATGTGCTACGAGAGCCCAA |
| Hd3a-qR | TGGCAGTTGAAGTAGACGGC |
| Hd1-qF | ACGACAACCGCATCGAAAAC |
| Hd1-qR | GCTGCTCACTCAGCATTGTG |
| Hd6-qF | TCACCGTTCAATGGGGTGAG |
| Hd6-qR | CGTGGGGTACAGCACTTTGA |
| Ghd7-qF | GAGCTTGAACCCAAACACGG |
| Ghd7-qR | CCTGATCAGCTTCTTTGGCG |
| Ehd1-qF | ACACGGAGCGACTTTGTCAT |
| Ehd1-qR | TGCACTCTGAGCCACTTGAG |
| actin-qF | CTGGTATTGCTGACCGTAT |
| actin-qR | GTTGGAAGGTGCTAAGGGA |

The underscore indicates the joint of the primer for homologous recombination.
